# Drift of the Earth’s Principal Axes of Inertia from GRACE and Satellite Laser Ranging Data

**DOI:** 10.3390/rs12020314

**Published:** 2020-01-18

**Authors:** José M. Ferrándiz, Sadegh Modiri, Santiago Belda, Mikhail Barkin, Mathis Bloßfeld, Robert Heinkelmann, Harald Schuh

**Affiliations:** 1 UAVAC, University of Alicante, Campus de San Vicente, 03080 Alicante, Spain; 2 GFZ German Research Centre for Geosciences, 14473 Potsdam, Germany; 3 Institute for Geodesy and Geoinformation Science, Technische Universität Berlin, 10623 Berlin, Germany; 4 Image Processing Laboratory (IPL)—Laboratory of Earth Observation (LEO), University of Valencia, 46980 Valencia, Spain; 5 Bauman Moscow State Technical University, 1 Bld., 5, 2nd Baumanskaya str., Moscow 105005, Russia; 6 Deutsches Geodätisches Forschungsinstitut Technische Universität München (DGFI-TUM), 80539 Munich, Germany

**Keywords:** earth gravity mission, GRACE, Satellite Laser Ranging, principal axes of inertia, earth rotation

## Abstract

The location of the Earth’s principal axes of inertia is a foundation for all the theories and solutions of its rotation, and thus has a broad effect on many fields, including astronomy, geodesy, and satellite-based positioning and navigation systems. That location is determined by the second-degree Stokes coefficients of the geopotential. Accurate solutions for those coefficients were limited to the stationary case for many years, but the situation improved with the accomplishment of Gravity Recovery and Climate Experiment (GRACE), and nowadays several solutions for the time-varying geopotential have been derived based on gravity and satellite laser ranging data, with time resolutions reaching one month or one week. Although those solutions are already accurate enough to compute the evolution of the Earth’s axes of inertia along more than a decade, such an analysis has never been performed. In this paper, we present the first analysis of this problem, taking advantage of previous analytical derivations to simplify the computations and the estimation of the uncertainty of solutions. The results are rather striking, since the axes of inertia do not move around some mean position fixed to a given terrestrial reference frame in this period, but drift away from their initial location in a slow but clear and not negligible manner.

## 1 Introduction

As for any extended body, the Earth’s general motion, i.e., its orbital translation and its rotation, depends on a number of dynamical parameters associated to its mass distribution, particularly the center of mass (COM) and the inertia matrix, as well as on the gravitational field that controls the Earth’s interplay with external bodies, including either natural or artificial satellites. As the Earth undergoes continuous changes at a large variety of time scales, from its inner to outer components, all of those dynamical parameters do not have steady values, but vary with time [1]. Their variations are so small compared to the constant reference values that they could not be observed until recent years, triggered by many geodetic satellite missions and the development of the main space geodetic techniques. The most extended time series for such parameters are those providing the COM, to which the satellite laser ranging (SLR) technique is the most sensitive. During many years, SLR was also the main tool to determine the gravity field of the Earth, derived from an expansion of its gravitational potential in terms of spherical harmonics (SH) [2]. The determination of the Earth’s gravity field reduces to the determination of the coefficients of the spherical harmonics (SHC), also known as Stokes coefficients, until certain attainable degree and order. SLR had a decisive contribution to infer the early accurate, steady gravity field models, and also allowed determining the time variations of an increasing number of lower degree SHC over the last decades [3—6]. If the COM, given by the three first-degree SHC, plays a vital role for the Earth’s orbit in the solar system and the dynamics of all the spacecrafts orbiting around the Earth, regarding the Earth’s rotation, the most relevant parameters are the second-degree SHC, linearly related to the inertia matrix [7]. In most textbooks and treatises on dynamics, rotations are studied after reducing the inertia matrix to diagonal form, i.e., theories and solutions of rotational problems are usually referred to the system of principal axes of inertia (PAI)—either with origin at the COM or at other point fixed with respect to the body, depending on the case at hand. Theories of Earth rotation are not an exception, and they are traditionally developed with respect to a reference system whose axes are aligned with the Earth’s PAI. However, space geodetic techniques were unable to provide accurate solutions for the Earth’s PAI until recent years, due to the level of uncertainty of the second-degree Stokes coefficients. For instance, according to the Groten 2004 “best” estimates [8] of fundamental parameters of interest in astronomy and geodesy, the Earth’s axis of major inertia was aligned with the axis of figure (or pole-to-pole axis) and the other two laid on the equatorial plane, with the minor axis pointing to the longitude 14.92910° ± 0.00012° W. However, a few earlier interesting studies addressing aspects of the PAI time evolution from satellite-based gravity models had already been published, mainly related to the creation of a dynamical reference frame (e.g., ^[9])^.

The launch of the NASA/DLR Gravity Recovery and Climate Experiment (GRACE) mission in 2002, reinforced by other posterior geodetic missions, contributed remarkably to improving the accuracy and spatial-temporal sampling of the Earth’s gravity field [10]. The geopotential models with constant Stokes coefficients that integrate GRACE data were already capable of determining the small deviations of the Earth’s PAI out of the equator or the polar axis, whose magnitude is below 1 arcsecond (as). Table 2b of Chen and Shen’s work [11] gives a good picture of the situation before and after GRACE concerning the location of the PAI, as well as a quick comparison of the advances of models EGM2008 and EIGEN-05C relative to the 2004 status [12,13]. Besides, the GRACE mission allowed detecting time variations of the Earth’s gravity field with at least monthly temporal resolution [10,14,15], a main goal of the mission design. Therefore, at present, we can compute not only highly accurate mean positions of the PAI but also their time variations with similar time resolution. However, the time evolution of the PAI location has never been investigated since the GRACE launch, despite its clear relation to the Earth’s rotational dynamics. The closest topic seems to be the deviation between the figure and rotation axes, useful to estimate the so-called secular Love number, of which only mean values were computed [11]. One plausible explanation may arise from the difficulty of the numerical computation of the PAI, especially if the error analysis associated with it is considered. Leaving aside speculations, after the completion of the GRACE mission, we consider it is the right time to obtain this new information from its data and find the main features of the PAI evolution.

In this paper, we use an approximate analytical method introduced by Barkin and Ferrandiz in 2000 [16] for deriving the PAI oscillations due to the Earth’s elasticity yielding to the Moon and Sun attraction and its rotation. The changes of the Earth’s PAI due to tidal and centrifugal (pole tide) deformations are included in the background models used to derive the Stokes coefficients and thus are excluded from the solution obtained in this paper by applying the same method to the time series providing the variation of the second-degree Stokes coefficients. Among the different data series available, we have chosen two independent solutions to present the results derived from the GRACE-based SHC monthly values provided by the Centre for Space Research (CSR), as well as those derived from a SLR-based SHC series computed at the Deutsches Geodätisches Forschungsinstitut Technische Universität München (DGFI-TUM) [7], with weekly resolution, which are described in more detail below.

## 2 Methods and Data

### 2.1 Basic Equations

The fundamental equations used in this approach are basically the same as those employed in [16]. Therefore, we present a short summary of them with identical notation and refer to that paper for additional details. For a given reference frame, the unnormalized second-degree Stokes coefficients are related to the elements of the Earth’s matrix of inertia through Equations (1)-(4):

(1) $$\begin{array}{rr} J_{2}=-C_{20}=\frac{2C-A-B}{2mR^{2}}, & C_{22}=\frac{B-A}{4mR^{2}},\\ S_{21}=\frac{D}{mR^{2}},\;\;\;\;C_{21}=\frac{E}{mR^{2}}, & S_{22}=\frac{F}{2mR^{2}}, \end{array}$$

where *A, B*, and C are the inertia moments and D, E, and *F* are the products of inertia. The axes should be principal, and the Stokes coefficients (denoted with superscript *p*) hold the relations

(2) $$\begin{array}{rr} J_{2}^{p}=-C_{20}^{p}=\frac{2C_{p}-A_{p}-B_{p}}{2mR^{2}}, & C_{22}^{p}=\frac{B_{p}-A_{p}}{4mR^{2}},\\ S_{21}^{p}=\frac{D_{p}}{mR^{2}}=0,\;\;\;\;C_{21}^{p}=\frac{E_{p}}{mR^{2}}=0, & S_{22}^{p}=\frac{F_{p}}{2mR^{2}}=0. \end{array}$$

In the general case, the moments and products of inertia can be computed from the principal ones using the rotation matrix *M* = (*a_ij_*), which relates the coordinates *X*_*np*_ = (*x*, *y*, *z*) and *X_p_* = (*ξ, η, ζ*) of a point in an arbitrary, non-principal reference frame, and the principal one, respectively:

(3) $$X_{np}=MX_{p}.$$

The relations among them are

(4) $$\begin{array}{ll} A=A_{p}+\left(B_{p}-A_{p}\right)a_{12}^{2}+\left(C_{p}-A_{p}\right)a_{13}^{2}, & B=B_{p}+\left(A_{p}-B_{p}\right)a_{21}^{2}+\left(C_{p}-B_{p}\right)a_{23}^{2},\\ C=C_{p}+\left(A_{p}-C_{p}\right)a_{31}^{2}+\left(B_{p}-C_{p}\right)a_{32}^{2}, & D=\left(A_{p}-B_{p}\right)a_{22}a_{32}+\left(A_{p}-C_{p}\right)a_{23}a_{33},\\ E=\left(B_{p}-A_{p}\right)a_{31}a_{11}+\left(B_{p}-C_{p}\right)a_{33}a_{13}, & F=\left(C_{p}-A_{p}\right)a_{11}a_{21}+\left(C_{p}-B_{p}\right)a_{12}a_{22}. \end{array}$$

The computation of the principal moments of inertia and the rotation matrix can be performed by solving a cubic equation whose roots are the principal moments. The main steps of the procedure are presented in several references (e.g., [11,16,17]).

However, when the deviations between the non-principal and principal frames are small and second-order terms can be neglected, a simple analytical approximation up to the first order can be derived, which allows obtaining the rotation matrix in the form

(5) $$M=\left[\begin{array}{ccc} a_{11}≅1 & a_{21}≅\frac{F}{B_{0}-A_{0}} & a_{31}≅\frac{E}{C_{0}-A_{0}}\\ a_{12}≅\frac{F}{B_{0}-A_{0}} & a_{22}≅1 & a_{32}≅\frac{D}{C_{0}-B_{0}}\\ a_{13}≅\frac{E}{A_{0}-C_{0}} & a_{23}≅\frac{D}{B_{0}-C_{0}} & a_{33}≅1 \end{array}\right]$$

From those equations, it is straightforward to show that the poles of the principal axes of inertia on a sphere of radius *R* are given up to the first order by

(6) $$\begin{array}{lll} x_{\xi }≅R, & y_{\xi }≅\frac{RF}{B_{0}-A_{0}}, & z_{\xi }≅\frac{RE}{C_{0}-A_{0}},\\ x_{\eta }≅\frac{RF}{B_{0}-A_{0}}, & \;\;\;\;\;\;\;\;\;\;\;\;y_{\eta }≅R, & z_{\eta }≅\frac{RD}{C_{0}-B_{0}},\\ x_{\zeta }≅\frac{RE}{A_{0}-C_{0}}, & \;\;\;\;\;\;\;\;\;\;y_{\zeta }≅\frac{RD}{B_{0}-C_{0}}, & \;\;\;\;\;\;\;\;\;\;\;\;\;\;z_{\zeta }≅R. \end{array}$$

In the above equations, the superscript 0 indicates that the relevant parameter can be evaluated at a fixed time even though the remaining parameters usually vary with time and their actual values must be used.

### 2.2 Simplifying Computations: Introduction of an Auxiliary Terrestrial Reference Frame (ATRF)

The international terrestrial reference frame (ITRF) [18] or similar frames (such as JTRF, DTRF, and others [19,20]) are not close to the PAI frame. However, they can be transformed into a system close to it by merely performing a rotation of about 14.9° westwards around the third axis, similar to Groten’s “best” Stokes coefficients [8]. The resulting frame is called here auxiliary terrestrial reference frame (ATRF). The ATRF does not have to coincide with the PAI at any time, according to the above equations. The deviations are small enough to obtain the rotation matrix by deriving an analytical approximation up to the first order. The justification is the same as presented in [16] to relate the PAI frame of the Earth in an ideal undeformed state to the instantaneous PAI frame after its tidal deformation, where the following equations were derived:

(7) $$\begin{array}{lll} \;\;\;\;\;\;\;\;\;\;x_{\tilde{\xi }}=1\text{, } & y\tilde{\zeta }=\frac{1}{2}\frac{\text{Δ}S_{22}}{C_{22}^{0}} & z_{\tilde{\xi }}=\frac{\text{Δ}C_{21}}{2C_{22}^{0}-C_{20}^{0}},\\ x_{\eta }=-\frac{1}{2}\frac{\text{Δ}S_{22}}{C_{22}^{0}}, & y_{\eta }=1\text{, } & z_{\eta }=-\frac{\text{Δ}S_{21}}{2C_{22}^{0}+C_{20}^{0}},\\ x_{\zeta }=-\frac{\text{Δ}C_{21}}{2C_{22}^{0}-C_{20}^{0}}, & y_{\zeta }=\frac{\text{Δ}S_{21}}{2C_{22}^{0}+C_{20}^{0}} & \;\;\;\;\;\;\;\;\;\;\;\;\;\;\;\;\;\;\;\;\;\;\;z_{\zeta }=1. \end{array}$$

Let us remark that those equations are valid up to first order of approximation relative to *C*_2*m*_, *S*_2*m*_, provided that the Stokes coefficients are referred to the ATRF or any other frame close enough to the actual PAI frame at the relevant time. In relation to Equation (6), the superscripts 0 correspond to chosen reference values of the principal moments of inertia. For the sake of studying the time evolution of them, any choice is feasible, for instance, their values at the initial or other chosen time, or an average. Transforming the second-degree SHC through a rotation of angle *α* around the *O_z_* axis (in the auxiliary system *O_x_y_z_*) is straightforward since the zonal term remains unchanged and the tesseral and sectorial terms of each frame are related through

(8) $$\begin{array}{rr} C_{21}^{ATRF}=\cos(\alpha )C_{21}^{ITRF}+\sin(\alpha )S_{21}^{ITRF}, & S_{21}^{ATRF}=-\sin(\alpha )C_{21}^{ITRF}+\cos(\alpha )S_{21}^{ITRF},\\ C_{22}^{ATRF}=\cos(2\alpha )C_{22}^{ITRF}+\sin(2\alpha )S_{22}^{ITRF}, & S_{22}^{ATRF}=-\sin(2\alpha )C_{22}^{ITRF}+\cos(2\alpha )S_{22}^{ITRF}. \end{array}$$

In the precedent equations, each coefficient is identified with the superscript corresponding to its reference frame. In such a way, we can derive time series of the PAI location from any time series providing the second-degree Stokes coefficients. It is worth mentioning that, for the computation of the uncertainties of the pole locations from the Stokes coefficients formal uncertainties, Equation (8) can be applied as well, unlike when computing the PAI poles by numerical methods since the former relations are linear.

### 2.3 Rotations Providing the Principal Axes

We think it is more intuitive to express the transformation of the frames through three infinitesimal rotations *R_x_*, *R_y_*, *R_z_*, as done, e.g., by Belda et al. (2016) [21]. The differences (△*_x_*, △*_y_*, △*_z_*) between the coordinates (*ξ*, *η*, *ζ*) in the mean monthly principal axis frame *O_ξηζ_* and the coordinates (*x*, *y*, *z*) in the auxiliary system *O_xyz_* are related to those rotations by:

(9) $$\left[\begin{array}{c} \text{Δ}x\\ \text{Δ}y\\ \text{Δ}z \end{array}\right]=\left[\begin{array}{ccc} 0 & -R_{z} & R_{y}\\ R_{z} & 0 & -R_{x}\\ -R_{y} & R_{x} & 0 \end{array}\right]\;\;\;\left[\begin{array}{l} x\\ y\\ z \end{array}\right]$$

The rotations transforming the quasi-principal ATRF into the PAI reference frame are

(10) $$\begin{array}{rrr} R_{x}=-\frac{\text{Δ}S_{21}}{2C_{22}^{0}+C_{20}^{0}}, & R_{y}=-\frac{\text{Δ}C_{21}}{2C_{22}^{0}-C_{20}^{0}}, & R_{z}=\frac{1}{2}\frac{\text{Δ}S_{22}}{C_{22}^{0}}, \end{array}$$

where superscripts 0 denote the chosen reference value of the relevant parameter, and the neglected terms are of second order concerning the variations of the Stokes coefficients. Let us notice that each rotation is simply related to one of the former pole coordinates, namely

$$R_{x}=-y_{\zeta ,}\;\;\;\;R_{y}=-z_{\xi },\;\;\;\;R_{z}=y_{\xi }$$

### 2.4 Input Data and Outline of the Analysis

Using the above equations, the time evolution of the Earth principal axes of inertia with respect to ATRF, a westward rotated ITRF, together with their estimated uncertainties, can be computed from any of the available, accurate geopotential solutions providing time-varying Stokes coefficients of degree 2, i.e., *C*_2*m*_ and *S*_2*m*_. In this paper, we use two of those series. First, the GRACE RL06 time series of normalized second-degree SHC provided by CSR, which contain monthly mean estimates of those coefficients referred to ITRF. The basic meaning of the coefficients can be found in [22–24]. For the purpose of this paper, it suffices to indicate that the background models used for the GRACE RL06 data processing, e.g., solid Earth and ocean tides and ocean pole tide models, are not included in the released SHC values; however, the monthly mean of the atmosphere-ocean de-aliasing model, included in the GRACE background models, has been restored. Additional details can be found in the UT/CSR RL06 processing standard technical document [25]. The data used in this paper span from January 2002 to August 2018.

The second series is provided by the DGFI-TUM and the SHC are derived from SLR analysis of up to ten geodetic SLR satellites. The processing standards of this solution follow the IERS Conventions (2010) [26], as indicated in [7,15]. From this dataset, we use the degree-2 normalized Stokes coefficients ${\bar{C}}_{2m}$ and ${\bar{S}}_{2m}$ with their corresponding time tags and uncertainties, with weekly resolution, along the period from January 2000 to February 2018.

The outline of the computation flow is the following for each of the time series. First, we transform from normalized to unnormalized Stokes coefficients, using the standard approach presented, e.g., in Equations 6.2b and 6.3 of [26]. Then, we obtain the SHC after performing a rotation of angle *α* = -14.9286648815724558 degrees (corresponding to an initial value *S*_22_ = 0 of the CSR rotated SHC) of the reference frame. That rotation angle defines the chosen ATRF, which is not principal since the (2, 1) SHC do not vanish, but is close enough to the PAI to enable the application of the previous equations. Then, the location of the PAI at each data point is found by computing the three infinitesimal rotations given by Equation (10). These three rotation time series, for each of the mentioned input datasets, are then analyzed with an emphasis on the identification of trends. All the statistics and the linear fit functions are computed weighting the input data with their squared formal errors and using the MAPLE 17 package of Maplesoft, a division of Waterloo Maple Inc., Waterloo, Ontario [27].

## 3 Results

**Figure 1:**
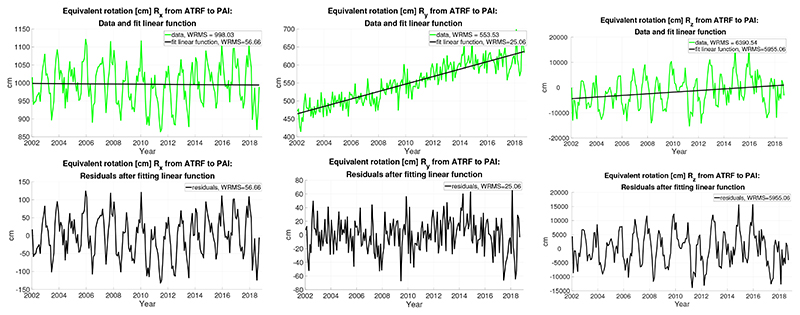
The results derived from the RL06 CSR Stokes coefficients with monthly resolution: (**top**) equivalent rotations [cm] *R_x_*, *R_y_*, and *R_z_* from ATRF to PAI frame (in green, from left to right) and fit linear functions (in black); and (**bottom**) the residuals are also given in [cm].

We present first the results corresponding to the RL06 CSR data. The rotations transforming the ATRF into the PAI frame at each date are not expressed in angular units but in equivalent centimeters on the Earth’s surface to give a quick picture of the magnitude of the pole axes evolution. The upper part of Figure 1 displays the three rotations *R_x_*, *Ry*, and *R_z_* (in green) and the first degree polynomials fit to them (in black); in the legend of each plot, there is a part that shows the WRMS (weighted root mean squared) of the date before and after the fit. The lower panel is made of another three plots that display the respective residuals. Notice that the vertical scales of the plots are different. The values of the WRMS statistics after the fit and the coefficients of the linear regression line, together with their formal errors (1*σ*), are shown in Table 1. The time origin is set to be the date JD 2000.0 (modified Julian date 51,544.5). All coefficients apart from the *R_x_* trend are larger than *3σ*, thus significant. The largest rotation is *R_z_*, around the *O_z_* axis of the ATRF, coincident with the ITRF *O_z_*. Its trend reaches 321.88 ± 86.78 cm/year on the Earth’s equator, and its direction is eastwards since it is positive (counterclockwise rotation, bringing the instantaneous *O_x_* closer to the ATRF *O_y_*). The trends of the other rotations are much smaller. The rotation *R_x_* around the *O_x_* axis is negative (clockwise), driving the ATRF *O_z_* axis towards the positive *O_y_* axis, with the smallest velocity of roughly -0.26 ± 0.82 cm/year. Finally, the *R_y_* rotation is positive (counterclockwise), moving *O_x_* and *O_z_* southwards (*O_z_* towards to the ATRF *O_x_*) by 10.40 ± 0.36 cm/year on the surface.

The first and third rotations also exhibit at first glance large, nearly seasonal variations. They might be attributed to the seasonal variations visible in the GRACE gravity fields or to deficiencies of the background models, at least to some extent. A preliminary Fourier analysis confirms that the main period of all the rotations is annual, although it also detects power at many other frequencies, such as those associated to the semi-annual, Chandler, and semi-Chandler periods, in general with noticeably smaller amplitudes and correspondingly larger formal errors. Furthermore, the monthly spacing of the data limits the temporal resolution attainable for the determination of periods and complicates the interpretation of the potential source of those signals. Therefore, we do not display any periodogram and leave the subject for further investigation.

**Figure 2:**
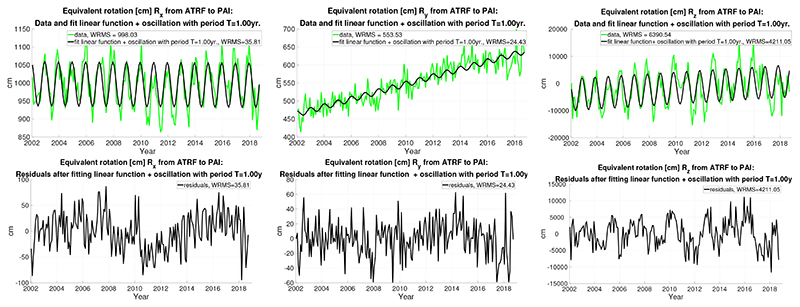
The results derived from the RL06 CSR Stokes coefficients with monthly resolution: (**top**) equivalent rotations [cm] *R_x_*, *R_y_*, and *R_z_* from ATRF to PAI frame (in green, from left to right) and fit linear functions plus annual oscillations (in black); and (**bottom**) the residuals are also given in [cm].

Despite that, as the annual signal looks clear and prominent, we present also the results of fitting a linear function jointly with an annual harmonic oscillation to the three rotations. Figure 2 (top) shows the plots of *R_x_*, *R_y_*, and *R_z_* displayed in green in analogy to Figure 1, as well as a linear function plus an annual oscillation fitted to *R_x_*, Ry, and *R_z_*, in black color. Please mind again the different scales of the axes. It can be seen that the residuals are smaller than in the previous case as shown in Table 2 (similar to Table 1). It illustrates to what extent the inclusion of the annual oscillation in the fit decreases the WRMS of *R_x_*, *R_z_*, and less the WRMS of *R_y_*. Besides, the coefficients of the annual fit to the latter rotation are much smaller than the other two, as shown in the last columns of Table 2. The trends of *R_x_* and *R_y_* are almost indifferent to the ones in Table 1, whereas the *R_z_* trend differs by about 6% between the two fits.

**Figure 3:**
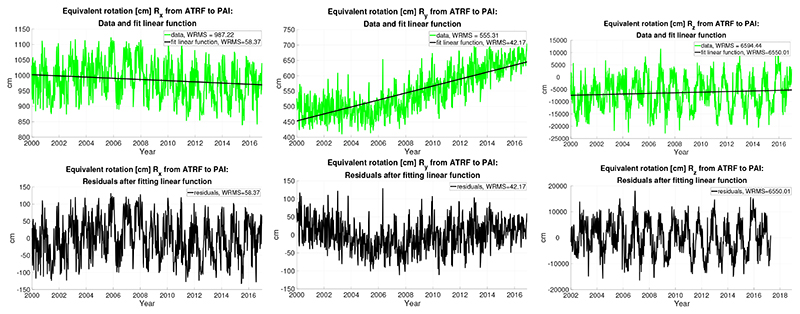
The results derived from the SLR DGFI-TUM Stokes coefficients with weekly resolution: (**top**) equivalent rotations [cm] *R_x_*, *R_y_*, and *R_z_* from ATRF to PAI frame (in green, from left to right) and fit linear functions (in black); and (**bottom**) the residuals are also given in [cm].

Next, we present the results obtained after performing the same computations using as input data the weekly DGFI-TUM time series derived from SLR analysis [15]. Figure 3 is equivalent to Figure 1 for the former RL06 dataset and thus does not require description. Let us recall that the DGFI-TUM series do not cover the same time interval as the CSR one: it starts in 2000, two years earlier, and ends a few months earlier in 2018. However, we prefer to use the whole length of each series since we are specially interested in trends and the series are rather short, the CSR one being limited by the operation period of the GRACE satellites. Again, the vertical scales of the plots are subject to change, as in the previous cases.

The statistical results derived from the SLR DGFI-TUM Stokes coefficients with weekly resolution are shown in Table 3. In this case, we do not include the results of fitting a straight line plus an annual oscillation for the sake of brevity. To discard that the higher temporal resolution of the DGFI-TUM input data might cause some distortion of the results, we have also analyzed a smoothed version of them with a monthly resolution similar to the CSR data and found that there is no relevant difference in the results. The resulting drifts are $R_{x}^{\prime }=-1.96\pm 0.60$, $R_{y}^{\prime }=11.42\pm 0.39$, $R_{z}^{\prime }=120.55\pm 71.46$ cm/year, very close to the weekly ones.

## 4 Discussion

First, we want to point out that the values of the biases of the former fits are not meaningful in our approach since the ATRF can be defined arbitrarily, with the only condition of having the small departure from the time-varying PAI frames. Therefore, we focus on the remaining parameters, particularly on the trends. It may be somehow striking that the trends of the *R_z_* rotation are at least one order of magnitude larger than the ones of the other two rotations. That is because the value is obtained after a division by *C*_22_ according to Equation (10), while in the other two the denominators have the order of magnitude of *C*_20_. Consequently, the motion of the first two axes of inertia along the equator is expected to be much larger than the shift of the *O_z_* axis. An analogous feature appeared in the tidal periodic perturbations of the Earth’s PAI computed by Barkin and Ferrándiz [16], where the maximum amplitude for the third axis (corresponding to the fortnightly perturbation) was about 19 km, whereas, for the other axes, the oscillation at the same period is only of about 19 m, as displayed in Table 3, Row 11 of their work. In other words, when computing the Earth’s PAI from gravity observations, it is much more straightforward to determine the equator through the equatorial bulge (large *C*_20_ value) than to discriminate between locations on the equator (smaller *C*_22_ value).

We proceed now to compare the results obtained from the two involved datasets, based on the UT/CSR GRACE RL06 and the DGFI-TUM SLR solutions. The respective trends are rearranged in Table 4, where the columns refer to the individual rotations. The values of the annual trends (“drifts”) fit to the dataset are indicated in the first row (CSR or DGFI-TUM), and the 95% confidence intervals (“CI95”) are displayed in the adjacent columns to the right. It can be seen that the values of the *R_y_* trends are very close, but the other two look quite different. However, the respective CI95 of the latter two do overlap, and therefore we can conclude that the trends of *R_x_* and *R_z_* do not differ significantly. As for *R_y_*, their two CI95 do not overlap, but the distance between them is only of 1 mm over about 10 cm, and thus a slight increase of the level of significance would result in overlapping. In fact, when the CSR based results are compared to the smoothed DGFI-TUM ones, the corresponding CI95 overlap.

Therefore, the results are robust enough to be significant and can be considered a physical feature of the Earth change, not an artifact. The last column of Table 4 contains the mean value of the two trends, which may be assumed to be a preliminary reference value of the linear trend associated with each rotation, i.e., the annual drift of the relevant axis. Although some of the values are very small, all of them are above the accuracy and stability requirements asked for by GGOS, the Global Geodetic Observing System of the International Association of Geodesy (IAG), to the reference frames and related parameters, namely 1 mm in position and 0.1 mm/year in velocity.

The small differences found between the two solutions may arise from some differences in background models or processing strategies. As can be seen in Figures 1 and 3, there is a clear trend in the *R_y_* component in both solutions. Nevertheless, the trend seems to accelerate around the years 2005–2006 of the DGFI-TUM time series. The difference in the *C*_21_ and *S*_21_ derived rotation time series might be caused by the different handling of the mean pole in the CSR RL06 and the DGFI-TUM solution. Using different mean pole model could provoke a systematic long-term difference in the *C*_21_ and *S*_21_ time series [28–31]. The CSR RL06 solution is processed based on the new linear mean pole model, whereas the DGFI-TUM SLR time series is based on the conventional (cubic polynomial) mean pole model [26]. Our results provide a further example of the relevance of the choice of a mean pole model.

The magnitude of the *R_z_* rotation, which drives the other two axes eastwards along the equator at a velocity of about 2.2 m/year, is surprising since that motion has not been detected until now. However, the satellite-derived time-varying gravity field solutions have been available for not too many years and have never been examined for that purpose. If we consider other ways of identifying that motion, the analysis of the evolution of the Earth orientation parameters (EOP) is not as suitable as this approach (although the EOP may be affected by the PAI drift). In fact, most of the nutation theories are based on a symmetric Earth model; therefore, they are not sensitive at all to the rotation of the inertia axes A and B, lying almost on the equatorial plane. Similarly, most of the investigations on polar motion and length of day (or UT1) also neglect the terms corresponding to the Earth’s triaxiality and thus become insensitive to the motions of those inertia axes. An additional difficulty arises from the aliasing between the diurnal rotation (for instance UT1) and the node of the satellite orbits.

**Figure 4:**
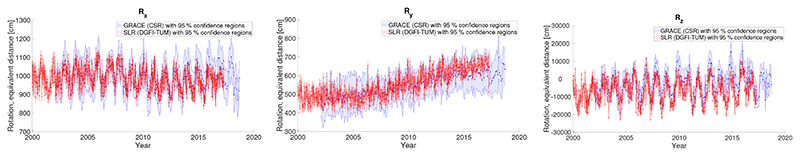
Joint plots of the rotations from ATRF to PAI frames derived from the RL06 CSR and SLR DGFI-TUM Stokes coefficients with their respective 95% confidence regions: (**left**) *R_x_*; (**middle**) *R_y_*; and (**right**) *R_z_*. Color code: CSR, curve in blue, confidence region in light blue; DGFI-TUM, curve in red, confidence region in light red. Units: cm (equivalent).

As additional evidence in support of our finding of the agreement of the two solutions, we complete this section with joint plots of the rotations computed from each dataset (displayed as a line) together with their CI95 (displayed as a colored area around the line). It can be visualized easily in Figure 4 that the solutions for each of the three rotations *R_x_*, *R_y_*, and *R_z_* are very similar across the entire time interval, which reinforces the hypothesis that the two solutions do not exhibit significant differences.

## 5 Conclusions and Outlook

The computation of the motion of the Earth’s principal axes of inertia using two different datasets for the time-varying second-degree Stokes coefficients, derived from GRACE and SLR solutions, shows a significant agreement, despite small differences in the processing standards and time interval or range of each solution—monthly and weekly, respectively. The most remarkable feature is that the determined principal axes of inertia of the Earth are clearly closely aligned to the ITRF axes or oscillating around certain “mean” equilibrium position, but exhibit non-negligible drifts, with magnitudes clearly exceeding the accuracy threshold of GGOS, the IAG Global Geodetic Observing System. The most remarkable detected motion drives the two nearly equatorial inertia axes eastwards, at a rate of 2.2 m/year. Besides, the axis of less inertia deviates away from the equator southwards at 11 cm/year, and the medium axis also moves southwards out of that plane at a smaller rate of 1 cm/year. The axis of major inertia follows the drifts of the other two.

This paper is intentionally limited to quantifying observational facts, but the physical causes of the drift of the Earth’s principal axes is still unknown, as well as its impact on other topics, e.g., Earth rotation. Getting more insight into these topics seems to be not an easy task, but different ideas or issues may emerge from future discussions about the topic, e.g. whether or not motion is due mainly to a sole cause, concerning external mass transport, changes in the Earth’s inner layers, tectonics, or a combination of processes. In this case, the geophysical budget could be closed to some extent. Another open question is whether these variations of the Earth’s inertia tensor might affect other processes of the Earth and could be related to, e.g., decadal or long-term EOP variations or other observed trends.

## Figures and Tables

**Table 1:** The results derived from the RL06 CSR Stokes coefficients with monthly resolution. WRMS after fitting a linear function to each, biases, and drifts of the equivalent rotations *R_x_*, *R_y_*, and *R_z_* from ATRF to PAI. Units are (cm) and (cm/year), time origin is 2000.0.

	WRMS [cm]	Bias [cm]	Drift [cm/year]
*R_x_*	56.66	999.12 ± 9.35	−0.26 ± 0.82
*R_y_*	25.06	442.92 ± 4.13	10.40 ± 0.36
*R_z_*	5955.06	−5029.83 ± 989.57	321.88 ± 86.78

**Table 2:** The results derived from the RL06 CSR Stokes coefficients with monthly resolution.WRMS after fitting a linear function and an annual oscillation to each, biases, drifts, and annual amplitudes of the equivalent rotations *R_x_*, *R_y_*, and *R_z_* from ATRF to PAI. Units [cm] and [cm/year], time origin 2000.0, null phase at the origin.

	WRMS [cm]	Bias [cm]	Drift [cm/year]	Amp. cos [cm]	A sin [cm]
*R_x_*	35.81	998.32 ± 5.95	−0.26 ± 0.82	58.80 ± 3.58	−19.32 ± 3.64
*R_y_*	24.43	442.67 ± 4.050	10.41 ± 0.35	7.86 ± 2.44	−0.21 ± 2.49
*R_z_*	4211.05	−4747.51 ± 703.91	302.93 ± 61.70	3408.96 ± 420.54	−4924.77 ± 428.73

**Table 3:** The results derived from the SLR DGFI-TUM Stokes coefficients with weekly resolution. WRMS after fitting a linear function to each, biases, and drifts of the equivalent rotations *R_x_*, *R_y_*, and *R_z_* from ATRF to PAI. Units are [cm] and [cm/year], time origin is 2000.0.

	WRMS [cm]	Bias [cm]	Drift [cm/year]
*R_x_*	58.37	1005.23 ± 3.25	−2.15 ± 0.39
*R_y_*	42.17	448.42 ± 2.78	11.78 ± 0.28
*R_z_*	6550.04	−7474.45 ± 436.28	120.073 ± 43.61

**Table 4:** Comparison of the linear trends of the rotations *R_x_*, *R_y_*, and *R_z_* obtained from the monthly CSR and weekly DGFI-TUM solutions. CI95 denotes the 95% confidence interval of the fit drift value. Units are cm/year.

	Drift (RL06)	CI95 (RL06)	Drift (DGFI-TUM)	CI95 (DGFI-TUM)	Mean Drift
*R_x_*	−0.26 ± 0.82	(−1.9,1.4)	−2.15 ± 0.39	(−2.9, −1.4)	−1.21
*R_y_*	10.40 ± 0.36	(9.7,11.1)	11.78 ± 0.28	(11.2,12.3)	11.09
*R_z_*	321.88 ± 86.78	(150.8,493.0)	120.07 ± 43.61	(34.5,205.7)	220.97
